# Novel interplay between JNK and Egfr signaling in *Drosophila* dorsal closure

**DOI:** 10.1371/journal.pgen.1006860

**Published:** 2017-06-19

**Authors:** Tatyana Kushnir, Sharon Mezuman, Shaked Bar-Cohen, Rotem Lange, Ze'ev Paroush, Aharon Helman

**Affiliations:** Department of Developmental Biology and Cancer Research, IMRIC, Faculty of Medicine, The Hebrew University, Jerusalem, Israel; University of California, Los Angeles, UNITED STATES

## Abstract

Dorsal closure (DC) is a developmental process in which two contralateral epithelial sheets migrate to seal a large hole in the dorsal ectoderm of the *Drosophila* embryo. Two signaling pathways act sequentially to orchestrate this dynamic morphogenetic process. First, c-Jun N-terminal kinase (JNK) signaling activity in the dorsal-most leading edge (LE) cells of the epidermis induces expression of *decapentaplegic* (*dpp*). Second, Dpp, a secreted TGF-β homolog, triggers cell shape changes in the adjacent, ventrally located lateral epidermis, that guide the morphogenetic movements and cell migration mandatory for DC. Here we uncover a cell non-autonomous requirement for the Epidermal growth factor receptor (Egfr) pathway in the lateral epidermis for sustained *dpp* expression in the LE. Specifically, we demonstrate that Egfr pathway activity in the lateral epidermis prevents expression of the gene *scarface* (*scaf*), encoding a secreted antagonist of JNK signaling. In embryos with compromised Egfr signaling, upregulated Scaf causes reduction of JNK activity in LE cells, thereby impeding completion of DC. Our results identify a new developmental role for Egfr signaling in regulating epithelial plasticity via crosstalk with the JNK pathway.

## Author summary

The developmental process of dorsal closure (DC) in *Drosophila* embryogenesis has provided many fundamental insights into conserved mechanisms controlling epithelial dynamics and cell migration, and therefore serves as a model for studying tissue morphogenesis and wound repair. DC is known to require the coordinated action of two signaling pathways: (i) the c-Jun N-terminal kinase (JNK) pathway, which is activated in the dorsal-most leading edge (LE) cells of the migrating epithelia; and (ii) the Decapentaplegic (Dpp) pathway, induced by the JNK pathway, which signals to neighboring lateral epidermis cells to drive the cellular changes required for DC. Here we uncover a new tier of regulation essential for DC, mediated by the Epidermal growth factor receptor (Egfr) pathway. Specifically, we demonstrate that Egfr signalling in the lateral epidermis suppresses the expression of the gene *scarface*, which encodes a secreted JNK antagonist. Through this regulatory switch the Egfr pathway facilitates JNK signalling in LE cells, ensuring full induction and activity of Dpp signaling that is pivotal for orchestrating the synchronized morphogenetic movements characteristic of DC. Our study thus identifies a novel mechanism of signal integration between the Egfr and JNK pathways, linking Egfr signalling to the core regulatory network controlling DC.

## Introduction

**Fig 1:**
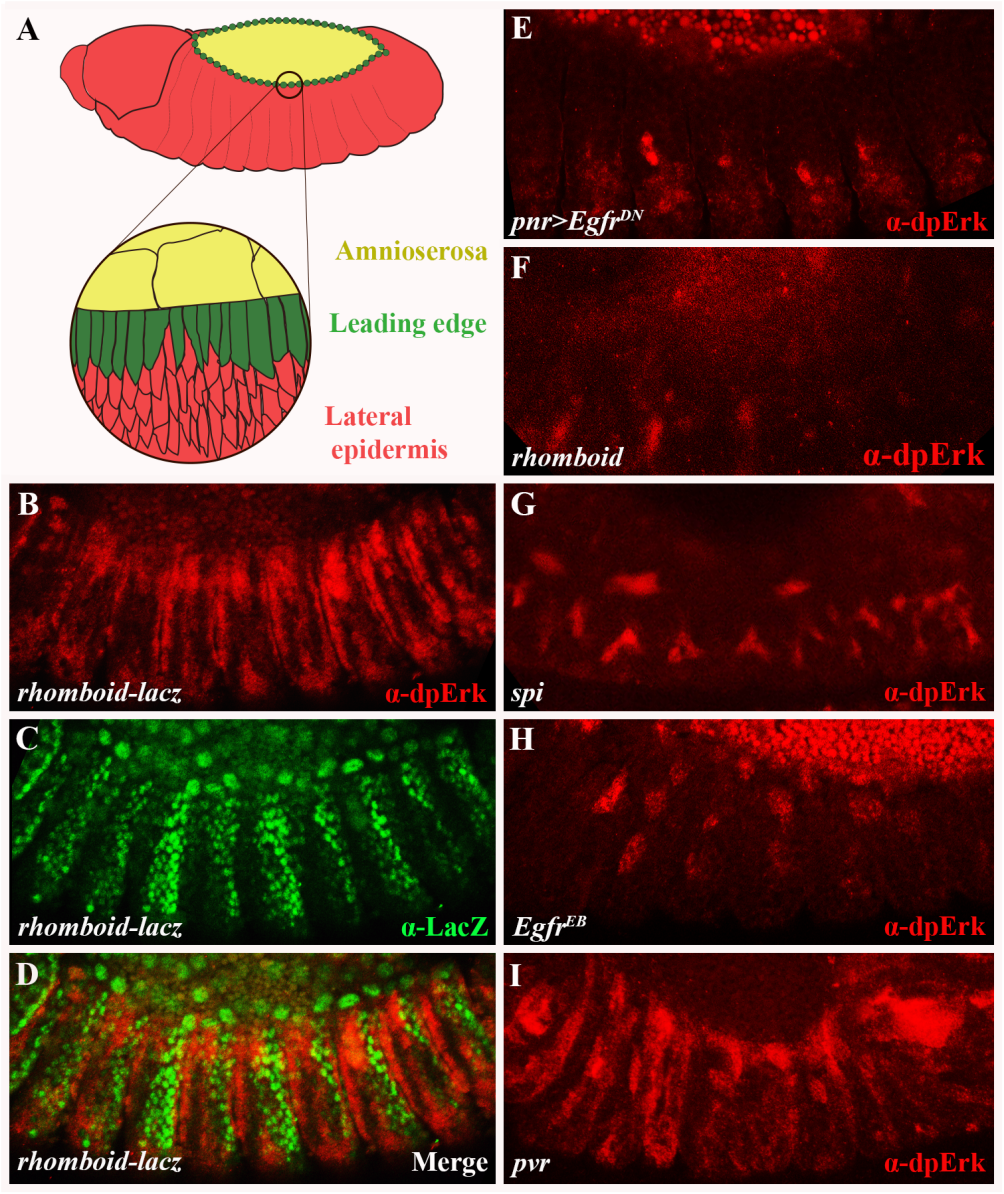
EGFR signaling takes place in the lateral epidermis of stage 13 embryos, at the time of dorsal closure. (A) A schematic lateral view of a stage 13 embryo (st13; 9:20–10:20 hours after egg lay). Demarcated are three groups of cells that participate in the process of DC: the amnioserosa (yellow), the dorsal-most row of ectodermal cells termed leading edge cells (green) and the adjacent lateral epidermis cells (red). (B-D) A *rhomboid*-*lacZ* enhancer-trap embryo co-stained for dpErk (B; red) and LacZ (C; green). (D) Merge. (B, D) dpErk staining is evident in the lateral epidermis. (C, D) dpErk staining borders on *lacZ* expression. (E-I) dpErk staining (red) is greatly reduced in the lateral epidermis of embryos expressing *pnr>Egfr*^*DN*^ (E), or in embryos mutant for *rhomboid* (F), *spi* (G) and *Egfr* (H), though not in *pvr* mutants (I). In (E) and (H), the signal in the AS is an artifact caused by auto-florescence. In this and all other Figures, embryos are at st13 and presented in lateral views, with anterior to the left and dorsal up, unless otherwise stated.

Epithelial sheet fusion and collective cell migration are key processes in normal development, wound healing and pathogenesis [1,2]. One system that has offered fundamental insights into the mechanisms controlling epithelial dynamics and cell migration is the embryonic process of dorsal closure (DC) in *Drosophila melanogaster*. In this developmental setting, two contralateral epithelial sheets from opposing sides of the embryo migrate and converge at the dorsal midline above the amnioserosa (AS), an extraembryonic epithelium tissue, thereby generating a continuous epidermis that seals a large dorsal hole (Fig 1A) [3]. Two cell signaling pathways, which act sequentially, drive and coordinate this process. Initially, c-Jun N-terminal kinase (JNK) pathway activity in the dorsal-most leading edge (LE) cells induces expression of the gene *decapentaplegic* (*dpp*), encoding the TGF-β/BMP family member Dpp [4–6]. Secreted Dpp subsequently triggers signal transduction in adjacent, ventrally located lateral epidermis cells, leading to the cell shape changes that are at the basis of the morphogenetic movements of the migrating epithelia [7,8]. Accordingly, embryos mutant for various components of the JNK signaling cascade or for constituents of the Dpp pathway fail to complete DC morphogenesis and consequently display dorsal-open phenotypes [9–11]. Thus, DC is an excellent experimental model system with which to identify and characterize the signaling events regulating complex movements of epithelial layers.

Spatiotemporal refinement of the expression of *dpp* and other JNK pathway target genes requires input by two negative feedback regulators, which are expressed in response to JNK signaling in LE cells [9]. One is Puckered (Puc), a dual specificity phosphatase that acts as an intracellular inhibitor of pathway activity by dephosphorylating JNK [12]. The other proposed JNK pathway antagonist is Scarface (Scaf), a secreted serine protease homologue that possibly acts by modifying the receptor mediating JNK signaling or an unknown extracellular signal [13,14]. Input from these two feedback inhibitors restricts JNK pathway activity in LE cells. It is not known, however, whether the expression of these negative regulators, or of other JNK pathway target genes in LE cells, is controlled only by JNK signaling, or whether other signaling pathways originating from adjacent epithelial cells might also contribute to this regulation.

Herein, we demonstrate an activity mediated by the Epidermal growth factor receptor (Egfr) pathway in lateral epidermis cells, that is pivotal for JNK function in the adjacent LE cells. Specifically, we identify a positive, cell non-autonomous input by the Egfr pathway, upstream of JNK signaling, into JNK pathway activity. We find that the mechanism underlying this effect involves the repression of *scaf* expression in lateral epidermis cells. Correspondingly, derepression of *scaf* in embryos defective in Egfr signaling causes a reduction in JNK activity in nearby LE cells. This leads to impaired expression of the JNK target gene *dpp*, reduced levels of Dpp effector responses, failure of cell elongation and, consequently, aborted DC resulting in a dorsal-open phenotype. Our results thus identify a novel intercellular crosstalk between the Egfr and JNK signaling pathways, shedding new light on DC regulation and potentially on other related processes involving synchronized cell movements and epithelial fusions [15].

## Results

### Egfr pathway activity is detectable in the lateral epidermis during dorsal closure

Intercellular signaling mediated by Receptor tyrosine kinases (RTKs) is essential for multiple patterning events during *Drosophila* oogenesis, embryogenesis and adult development [16–19]. We therefore reasoned that RTK-dependent signaling might also play unknown roles during the process of DC. Hence, we immunostained embryos for active mitogen-activated protein kinase/extracellular signal-regulated kinase (MAPK/Erk) as readout for pathway activity [20–22]. Doubly phosphorylated MAPK/Erk (dpErk) was detectable at the time of DC initiation in the lateral epidermis, a region where RTK activity has not been explored before (Fig 1A and 1B).

Several lines of evidence indicate that MAPK/Erk is activated in this region specifically in response to Egfr signaling. First, dpErk staining borders on the striped domains of *rhomboid* expression (determined using a *rhomboid-lacZ* enhancer trap line; Fig 1B–1D); this gene encodes the serine protease that is both necessary and sufficient to trigger Egfr pathway activity, and whose expression pattern forecasts Egfr pathway activation [22]. Second, activation of MAPK/Erk is significantly reduced in embryos in which *pannier* (*pnr*)-Gal4 [23] drives the expression of a dominant-negative form of Egfr (*Egfr*^*DN*^), thereby blocking Egfr function selectively in the lateral epidermis and the LE cells (Fig 1E and S1 Fig). Third, a reduction in dpErk staining is also evident in *rhomboid* mutants, as well as in embryos mutant for *spitz* (*spi*), which encodes a member of the TGF-α family of Egfr ligands [24], and for *Egfr* (Fig 1F–1H), though not in mutants for *pvr*, the gene encoding *Drosophila* Platelet-derived growth factor/Vascular endothelial growth factor receptor (PVR) (Fig 1I). Taken together, these results show that signaling via the Egfr pathway takes place in the lateral epidermis during the time of DC.

### Egfr pathway activity is required for proper dorsal closure

**Fig 2:**
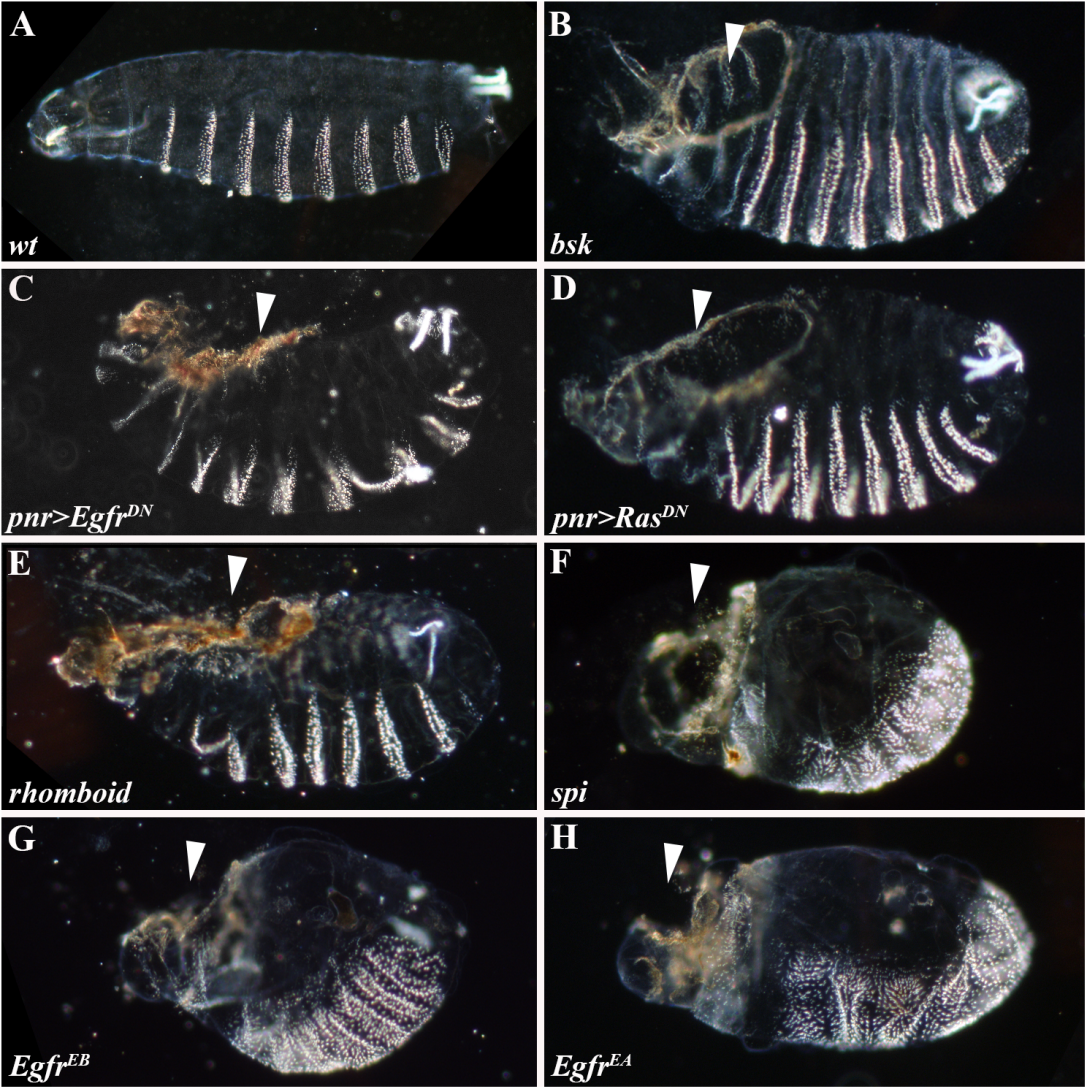
Dorsal closure is defective in embryos deficient for Egfr signaling. (A-H) Cuticle preparations. (A) Wild-type cuticle; note the complete closure of the epidermis on the dorsal side. (B) *bsk* mutant embryo; note the characteristic dorsal-open phenotype (arrowhead). (C-H) Lack of functional Egfr signaling leads to the formation of dorsal-open holes (arrowheads), a phenotype typically associated with JNK pathway mutants (cf. B). Egfr pathway activity was compromised by *pnr>Gal4*-driven ectodermal expression of *Egfr*^*DN*^ (C) or *Ras*^*DN*^ (D), or in *rhomboid* (E), *spi* (F) and allelic *Egfr* (G-H) mutant embryos.

To establish the functionality of Egfr signaling in the lateral epidermis, we examined the effects caused by the loss of Egfr-mediated signal transduction on different aspects of the closure process. Analyses of cuticle preparations revealed that embryos expressing dominant-negative forms of Egfr or Ras (*Ras*^*DN*^) via the ectodermal *pnr*-Gal4 driver (Fig 2C and 2D, respectively), as well as embryos mutant for *rhomboid*, *spi* or various alleles of *Egfr* (Fig 2E–2H), fail to complete DC and exhibit dorsal-open phenotypes (white arrows; cf. wild-type in Fig 2A), characteristic of mutants in genes encoding components of the JNK pathway [e.g., *basket* (*bsk*), encoding *Drosophila* JNK; Fig 2B] [7,25].

To quantify the proportion of embryos that fail to complete closure due to defective Egfr signaling, we scored the percentage of *Ras*^*DN*^- and *Egfr*^*DN*^-expressing embryos that completed DC by stage 16 (st16; 13–16 hours after egg lay), when the process normally ends (S2B Fig). In order to unambiguously segregate incomplete DC phenotypes from other secondary effects seen in Egfr-deficient embryos, such as head involution defects, we demarcated the LE cells using the *puc-lacZ* enhancer trap line, which concurrently also enabled the unequivocal identification of the embryonic genotypes scored (S2A and S2B Fig) (see Materials and Methods). We find that by st16, 92% of control embryos completed the course of DC, whereas only 47% and 39% of embryos expressing *Ras*^*DN*^ or *Egfr*^*DN*^ finalized closure, respectively, confirming that Egfr signaling plays a significant role in DC regulation (S2C Fig).

**Fig 3:**
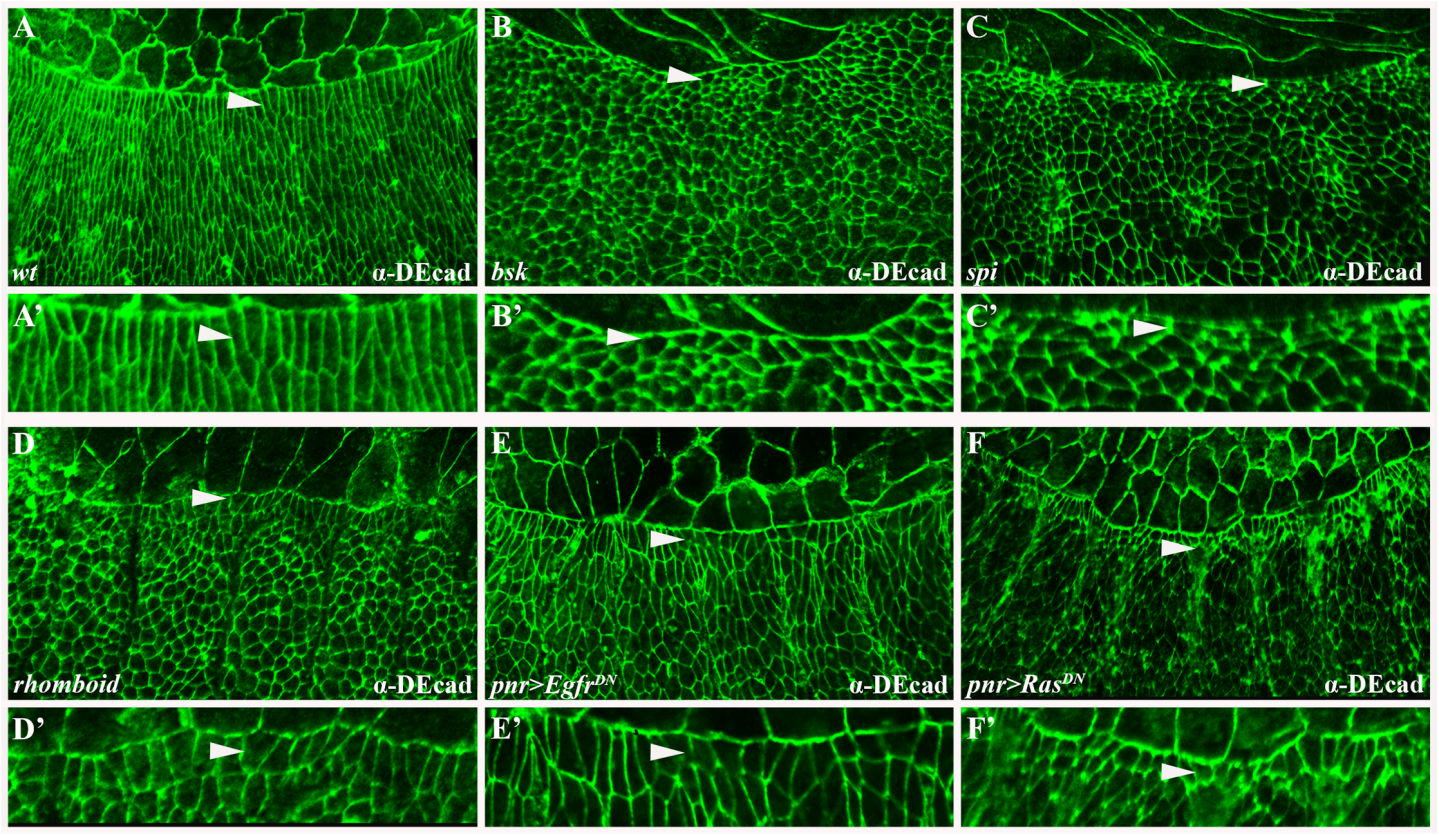
Functional Egfr signaling is required for the cell shape changes that occur during dorsal closure. (A-F) Embryos stained for DE-cadherin (green) to outline cell membranes. Corresponding primed panels (A’-F’) show magnified views of the regions marked with arrowheads. (A) Wild-type embryo. At this stage, both LE cells and cells in the lateral epidermis elongate and stretch along the D/V axis. (B) Elongation failure typifies *bsk* and other mutant embryos defective in JNK pathway signaling. (C-F) Deficiency in Egfr signaling in embryos mutant for *spi* (C) or *rhomboid* (D), as well as in those expressing *pnr>Egfr*^*DN*^ (E) or *pnr>Ras*^*DN*^ (F), leads to failure of epidermal cells to elongate. Instead, they remain polygonal, thus phenocopying *bsk* mutants (B).

Consistent with the cuticular defects, the consequences of loss of Egfr-mediated signal transduction are also apparent at the cellular level. Normally, LE cells of the advancing epidermis elongate along the dorsoventral (D/V) axis upon DC initiation. Subsequently, a similar elongation of lateral epidermis cells is observed in more ventral locations, as reflected by DE-Cadherin immunostaining that outlines cell membranes (Fig 3A and 3A’) [26,27]. In contrast, mutations in various constituents of the Egfr pathway, as well as ectodermal *Egfr*^*DN*^ or *Ras*^*DN*^ expression, prevent epithelial cell elongation to a large extent (Fig 3C–3F’, arrowheads; quantification in S3 Fig). Instead, many cells remain polygonal in shape, as do analogous cells in *bsk* mutant embryos (Fig 3B and 3B’; arrowheads) [28,29].

Collectively, our findings identify a requirement for functional Egfr signaling in the process of DC, which is already apparent at the level of the cell shape changes that normally occur during early DC.

### Egfr signaling is required for full induction and activity of Dpp signaling

**Fig 4:**
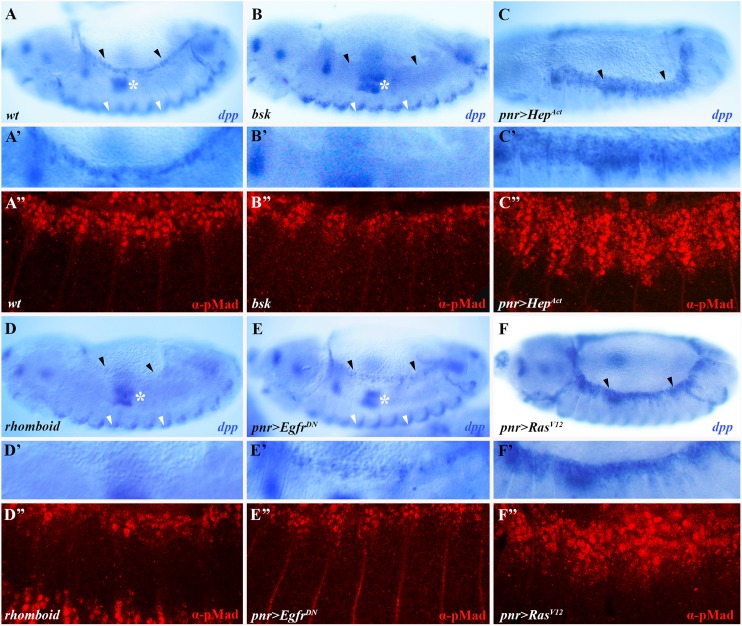
Egfr signaling is positively required for the full expression of the JNK pathway target gene *dpp* and for Mad phosphorylation. (A-F) Lateral (A, B, D, E) or dorsolateral (C, F) views of embryos hybridized using a digoxigenin-labeled RNA probe for *dpp* (blue). (A’-F’) Corresponding magnified views of the regions marked by black arrowheads in panels (A-F). (A”-F”) show embryos stained for pMad (red). (A-A”) Wild-type embryo showing the normal *dpp* (A-A’) and pMad (A”) patterns. Levels of *dpp* and pMad are reduced in *rhomboid* mutants (D-D”), as well as in embryo expressing *pnr>Egfr*^*DN*^ (E- E”). Conversely, both expand in embryo expressing *pnr>Ras*^*V12*^ (F- F”). These effects largely phenocopy loss- or gain-of-function JNK signaling (*bsk* mutant and *pnr>Hep*^*Act*^ embryo in B-B” and C-C”, respectively).

Dpp signaling is known to coordinate the morphogenetic movements during DC [8]. Considering the impaired cell shape changes observed in embryos defective in Egfr signaling (Fig 3), we next assessed the expression of the JNK pathway target *dpp* in LE cells of *rhomboid* mutants, as well as in embryos expressing *Egfr*^*DN*^ in the ectoderm, finding that it is reduced in both genotypes (black arrowheads in Fig 4D and 4E, respectively; cf. wild-type *dpp* expression in Fig 4A; S4 Fig) [6,7]. Noteworthy, in both genetic backgrounds JNK-independent *dpp* expression is unaffected, for example in the visceral mesoderm and lateral ectoderm (white asterisks and arrowheads, respectively, in Fig 4A, 4D and 4E) [5,25]. Similar outcomes were observed when *Egfr*^*DN*^ was expressed in stripes, using *paired* (*prd*)-Gal4 (S5 Fig). Reciprocally, the *dpp* expression domain expands in embryos expressing *Ras*^*V12*^, a constitutively active form of Ras (Fig 4F; S4 Fig). These effects closely resemble those observed when JNK signaling is blocked or constitutively active, respectively (cf. *bsk* mutants or embryos expressing an active form of the JNK kinase, *Hep*^*Act*^, in Fig 4B and 4C respectively).

In Dpp-responding cells, activation of the Dpp receptor complex brings about the phosphorylation of the SMAD family member Mothers against *dpp* (Mad) [30]. We find that levels of phosphorylated Mad (pMad) fully mirror the changes in *dpp* expression levels in the different genetic backgrounds (Fig 4B''–4F''; cf. wild-type in Fig 4A''). To determine which cells are most affected by the loss of Egfr signaling, we utilized the *puc-lacZ* enhancer trap line to delineate LE cells. We find that pMad is detectable in LacZ-expressing LE cells in embryos expressing *Egfr*^*DN*^ and *Ras*^*DN*^ under *pnr*-Gal4 regulation, consistent with residual *dpp* expression in these cells (Fig 4D–4E’; S4 Fig). However, in these genetic backgrounds pMad staining is noticeably reduced in the lateral epidermis (S6 Fig).

Cumulative effects due to dysfunction of the *Egfr* signaling pathway at earlier stages of development could contribute to the dorsal open phenotypes observed in embryos defective in Egfr signaling. To specifically assess the impact of this pathway on dorsal closure, independently from earlier contributions, we used a temperature sensitive *Egfr* allele (*Egfr*^*SH2*^) [31]. We find that *Egfr*^*SH2*^ embryos shifted from the permissive to the restrictive temperature (i.e., from 18°C to 29°C) at the onset of dorsal closure (st12) develop dorsal open phenotypes and other characteristic dorsal closure defects (S7 Fig).

We conclude that, Egfr signaling is required for the full induction of *dpp* and for the phosphorylation of the downstream Dpp pathway effector molecule, Mad.

### The JNK pathway is epistatic to Egfr signaling

**Fig 5:**
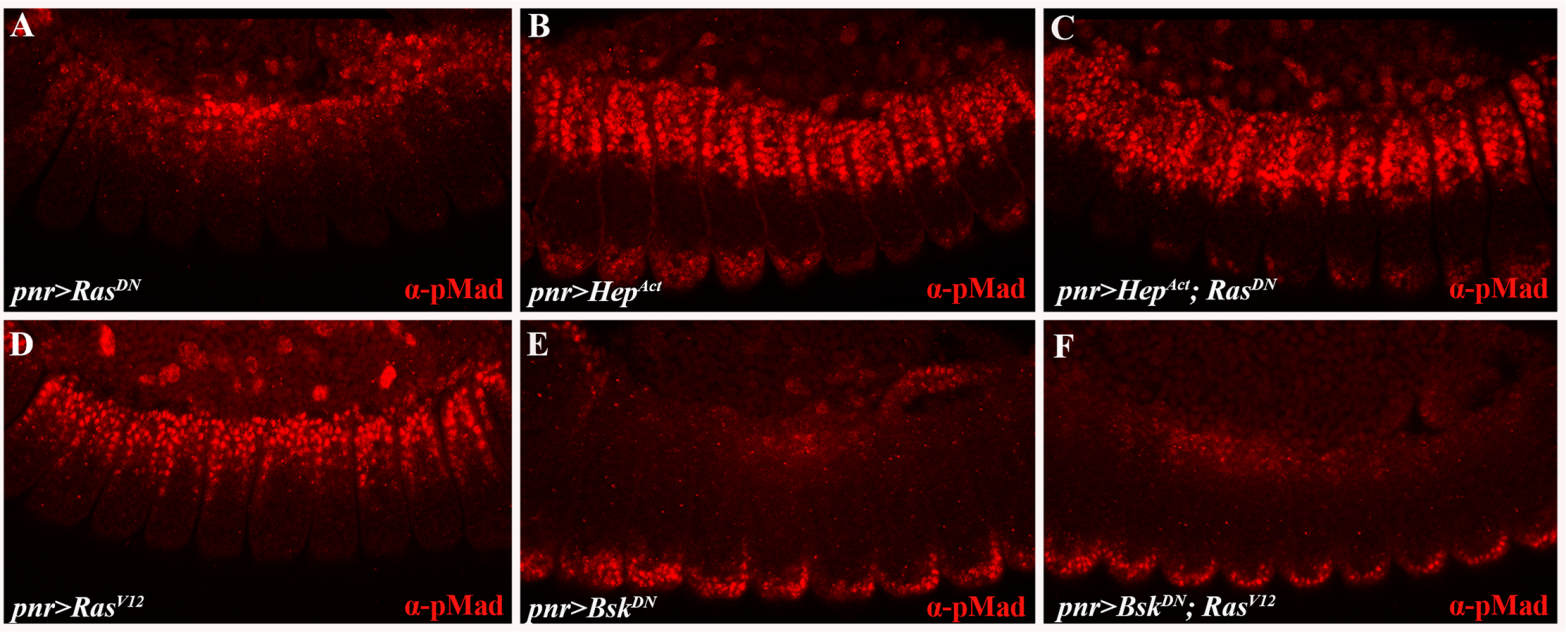
Egfr signaling acts upstream of the JNK cascade in dorsal closure. (A-F) Embryos stained for pMad (red). pMad staining decreases in embryos expressing *pnr>Ras*^*DN*^ (A) and expands in embryo expressing *pnr>Hep*^*Act*^ (B). In embryos co-expressing *pnr>Hep*^*Act*^; *Ras*^*DN*^, pMad staining expands (C). pMad staining expands in embryo expressing *pnr>Ras*^*V12*^ (D) and is largely reduced in embryo expressing *pnr>Bsk*^*DN*^ (E). In embryos co-expressing *pnr>Bsk*^*DN*^*; Ras*^*V12*^, pMad staining is also reduced (F). These results indicate that JNK signaling is epistatic to the Egfr pathway.

Given the phenotypic similarities between embryos defective in Egfr and JNK signaling, we next set out to determine the epistatic relationship between the two pathways using pMad staining. First, we assessed the ability of constitutive JNK pathway activation to suppress loss of Egfr signaling in the double combination of *Hep*^*Act*^ and *Ras*^*DN*^. When individually expressed, pMad staining is reduced in *pnr>Ras*^*DN*^ embryos compared to controls (Fig 5A; cf. Fig 4A''), and broader in those expressing *pnr>Hep*^*Act*^ alone (Fig 5B). Strikingly, the *Ras*^*DN*^-mediated reduction in pMad staining is fully suppressed by *Hep*^*Act*^ co-expression, with the pattern indistinguishable from that observed in embryos singly expressing *Hep*^*Act*^ (Fig 5C; cf. Fig 5B). This result indicates that the JNK pathway acts downstream of the Egfr pathway.

To further examine this issue we also undertook a reciprocal approach, by testing whether loss of JNK signaling suppresses constitutive activation of the Egfr pathway. Specifically, the pMad pattern expands in embryos expressing *Ras*^*V12*^ alone (Fig 5D; cf. Fig 4A'') and narrows in embryos expressing a dominant-negative form of Bsk (*Bsk*^*DN*^) by itself (Fig 5E). Markedly, pMad staining in combined *pnr>Bsk*^*DN*^; *Ras*^*V12*^ embryos resembles that seen in embryos expressing *Bsk*^*DN*^ alone (Fig 5F; cf. Fig 5E; S8 Fig). Taken together, these results demonstrate that the JNK cascade is epistatic to the Egfr pathway. Consistent with this conclusion, we find that MAPK/Erk activation in response to Egfr signaling is unaffected in *bsk* mutants as well as in embryos expressing *Hep*^*Act*^ (S9 Fig). Thus, our data identify a positive input by the Egfr pathway, acting upstream of JNK signaling, into the expression of a central JNK pathway target gene, *dpp*, and consequently into the phosphorylation of the key Dpp pathway effector, Mad.

### EGFR signaling is required for repression of *scarface*, a secreted inhibitor of the JNK pathway, in the lateral epidermis

**Fig 6:**
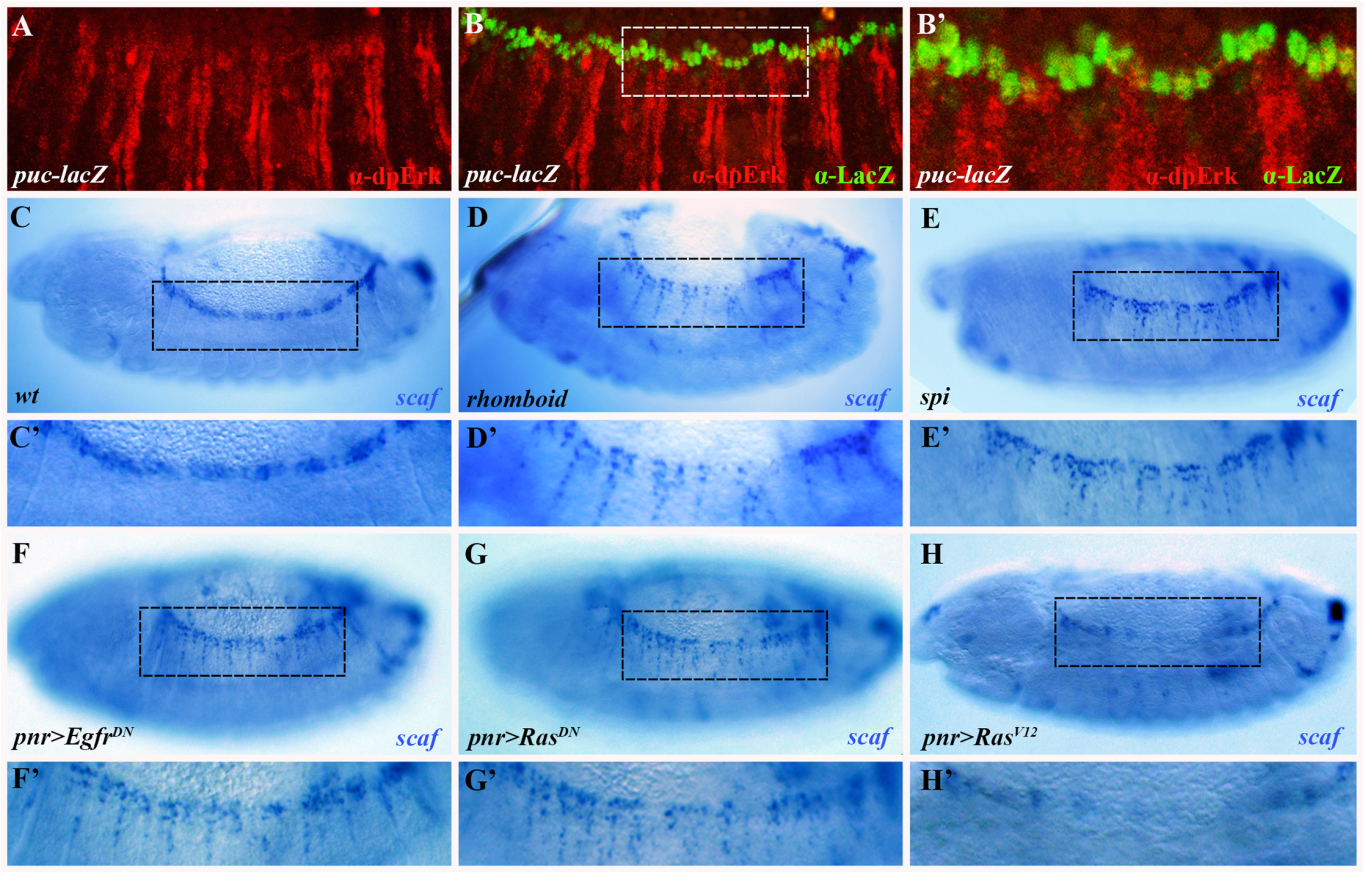
The Egfr pathway feeds into JNK signaling non-autonomously, via repression of the JNK antagonist *scarface*. (A-B’) *puc*-lacZ enhancer-trap embryo, double stained for dpErk (A; red) and for LacZ (B; green). (B’) Magnification of the region marked in B. Note that the dpErk and LacZ patterns do not overlap; instead, LacZ is detectable in LE cells whereas dpErk is observed in the adjacent lateral epidermis. (C-H) *In situ* hybridization for *scaf* using a digoxigenin-labeled RNA probe (blue). Corresponding primed panels (C’-H’) show magnified views of the marked rectangular regions. (C, C’) *scaf* is expressed in LE cells in wild-type embryos, but not in the lateral epidermis. (D-G) *scaf* expression expands ventrally in embryos with reduced Egfr pathway activity, such as *rhomboid* (D, D’) and *spi* (E, E’) mutants, and embryos expressing *pnr>Egfr*^*DN*^ (F, F’) and *pnr>Ras*^*DN*^ (G, G’). Conversely *scaf* expression declines in LE cells of embryos with augmented Egfr activity (*pnr>Ras*^*V12*^; H, H’).

The *rhomboid* and dpErk patterns (Fig 1D) indicate that the Egfr pathway is active throughout the dorsal ectoderm. To better understand the hierarchal link between the Egfr and JNK pathways, we co-stained embryos expressing *puc*-*lacZ*, a target of JNK signaling in the LE cells [12], for dpErk and LacZ. Surprisingly, dpErk staining does not overlap with the *puc-LacZ* pattern, and is evident only in the adjacent lateral epidermis (Fig 6A–6B’). The finding that dpErk is excluded from the LE demonstrates that the JNK and Egfr pathways are active in distinct cell types within the dorsal ectoderm, and therefore implies that the Egfr pathway influences JNK signaling cell non-autonomously.

We hypothesized that the indirect input by Egfr signaling in lateral epidermis cells into JNK activity in neighboring LE cells occurs either via induction of a secreted JNK agonist or by the suppression of a secreted JNK antagonist. Among known JNK pathway elements, the secreted JNK antagonist Scarface (Scaf) appeared to be an attractive candidate to perform this intermediary role. *scaf* is normally expressed in LE cells under JNK regulation (Fig 6C) [13,14]. However, reduced Egfr activity, in *rhomboid* and *spi* mutants as well as in embryos expressing *Egfr*^*DN*^ or *Ras*^*DN*^, results in ventral expansion of *scaf* expression in a striped configuration (Fig 6D–6G; cf. Fig 6C). By contrast, embryos expressing *Ras*^*V12*^ under *pnr-Gal4* regulation show a notable decrease in *scaf* expression even in LE cells (Fig 6H).

These data strongly suggest that Egfr signaling normally suppresses *scaf* expression, and provide a plausible explanation for the positive, non-autonomous effect of the Egfr pathway on JNK signaling in the LE. According to this scenario, in wild-type embryos Egfr signaling inhibits *scaf* expression in lateral epidermis cells, thereby blocking its secretion and antagonistic effect on JNK signaling in neighboring LE cells. Under *Egfr* loss-of-function conditions, *scaf* is up-regulated in lateral epidermis cells, leading to reduced JNK activity in LE cells. Accordingly, *scaf* expression in the LE itself is reduced relative to normal embryos (Fig 6D–6G’; cf. Fig 6C), and thus responds to the loss of *Egfr* signaling like other JNK pathway targets, such as *dpp*.

**Fig 7:**
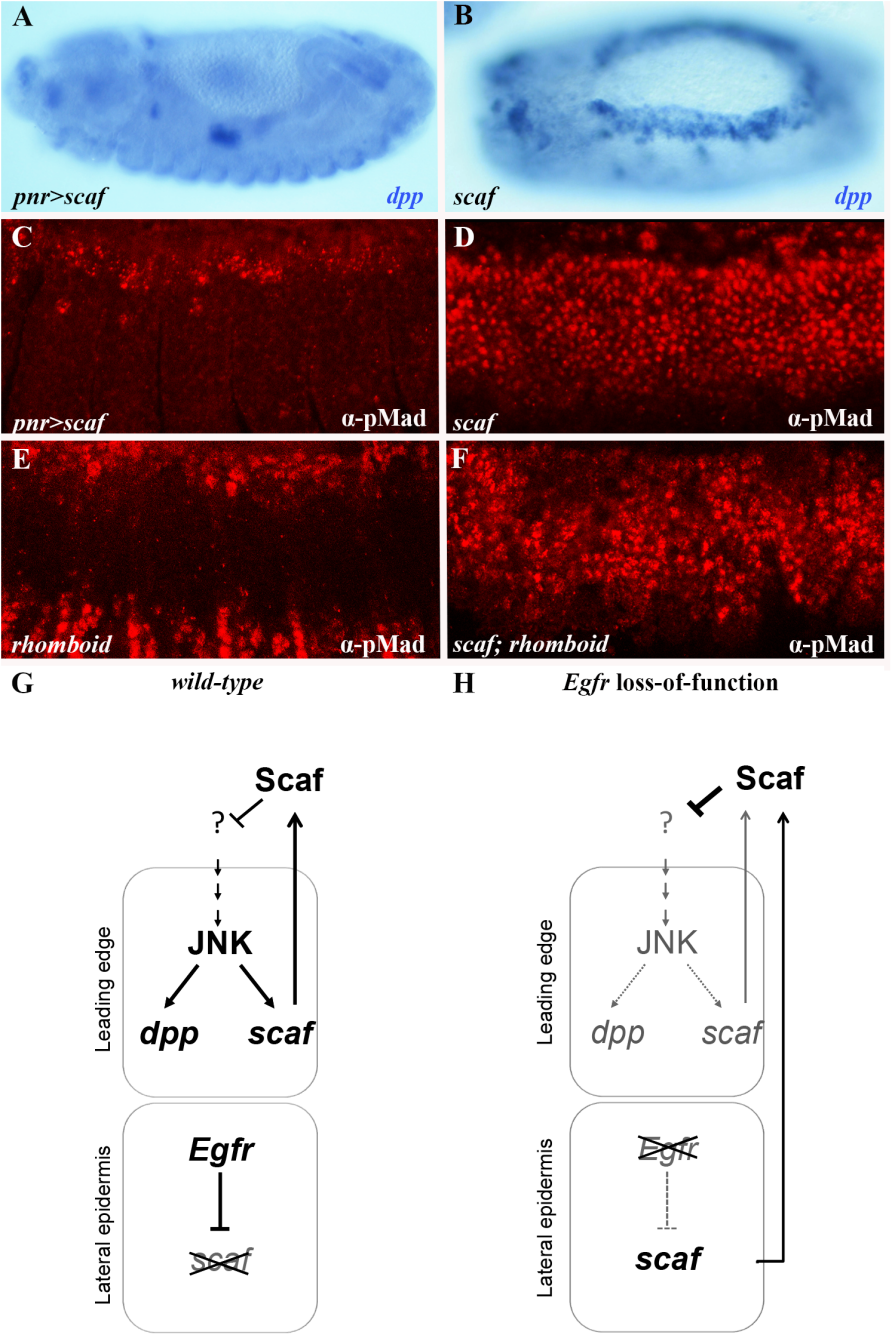
Over-expression of *scarface* mimics the loss of Egfr pathway activity. (A-B) Embryos hybridized using a digoxigenin-labeled RNA probe for *dpp* (blue). Over-expression of *scaf* brings about a reduction in *dpp* expression (A) whereas *dpp* expression expands in *scaf* mutant embryos (B), similarly to loss- and gain-of-function mutations in the Egfr pathway, respectively. (C-F) Embryos stained for pMad (red). (C, D) In keeping with *dpp* expression levels, pMad staining decreases upon *scaf* over-expression (C), and is augmented in a *scaf* mutant (D). (E, F) Although pMad staining is reduced in *rhomboid* single mutant embryo (E), it expands in embryo doubly mutant for *scaf* and *rhomboid* (F), as in *scaf* single mutant (D), indicating that *scaf* is epistatic to *Egfr* signaling. (G-H) Model showing how Egfr signaling in the lateral epidermis positively and non-autonomously contributes to JNK pathway activity in LE cells and to DC. (G) The Egfr pathway normally acts in the lateral epidermis to prevent expression of the JNK antagonist, *scaf*, thus supporting maximal JNK activity in LE cells. (H) When Egfr signaling is defective, deregulated Scaf subsequently attenuates functional JNK signaling in LE cells, thus hindering the process of DC. Bold text and arrows/bars indicate normal levels of gene expression and regulation, whereas gray fonts designate abnormally lower levels of expression and regulation, respectively.

To establish more directly how *scaf* deregulation influences JNK pathway output, we assessed *dpp* transcription and Mad phosphorylation in embryos over-expressing *scaf* or in *scaf* mutants. We find that *scaf* over-expression in the ectoderm obstructs *dpp* expression (Fig 7A; cf. wild-type in Fig 4A) and reduces Dpp pathway activity as reflected by immunostaining for pMad (Fig 7C; cf. wild-type in Fig 4A''), in a manner resembling *Egfr* loss-of-function backgrounds in which *scaf* is derepressed (Fig 4D–4E''). Conversely, both *dpp* expression and the pMad domain expand in *scaf* mutants (Fig 7B and 7D). These observations support a role for Scaf as a suppressor of Dpp signaling in the dorsal ectoderm.

The *scaf* over-expression and loss-of-function phenotypes closely parallel those observed for *Egfr* loss-of-function (Fig 4D–4E'') and RTK constitutive activation (Fig 4F and 4F''), respectively. To establish a regulatory link between Egfr signaling and *scaf*, we stained embryos, singly or doubly mutant for *rhomboid* and *scaf*, for pMad. The pMad domain broadens in *scaf* mutants (Fig 7D) and narrows considerably in *rhomboid* mutant embryos (Fig 7E). Importantly, embryos doubly mutant for *rhomboid* and *scaf* show an expanded pMad pattern (Fig 7F), similarly to embryos mutant for *scaf* alone (Fig 7D). These results indicate that Egfr signaling acts as an upstream negative regulator of *scaf* in the lateral epidermis cells. Thus, in the absence of *scaf*, JNK activity and consequent Dpp pathway activity is robust whether or not the Egfr pathway is functional. Collectively, our data indicate that Egfr-mediated suppression of *scaf* in lateral epidermis cells is required for full JNK signaling activity in LE cells, accounting for how Egfr activity affects JNK signaling in nearby cells and explaining why the Egfr pathway is required for the completion of the DC process (Fig 7G and 7H).

**Fig 8:**
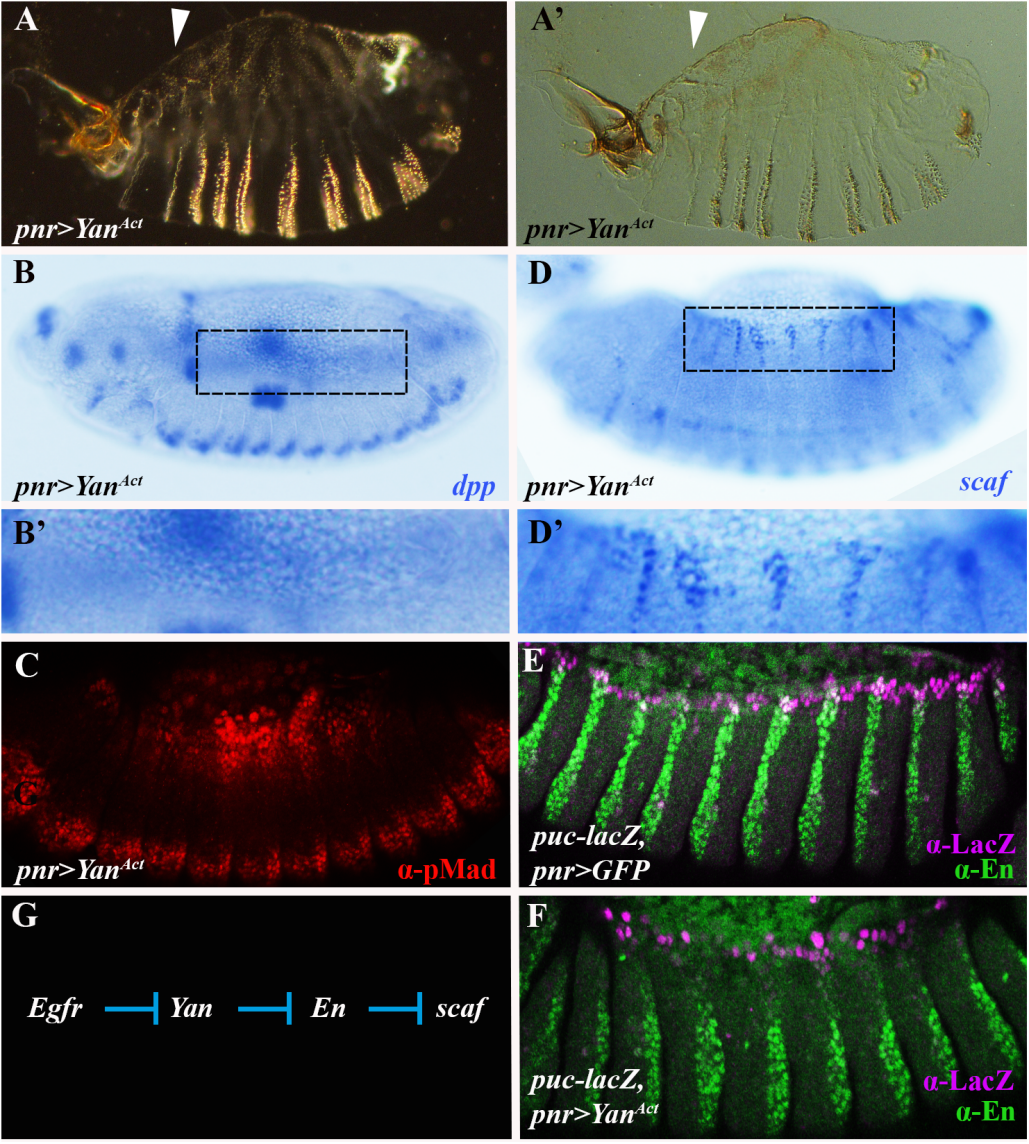
The Egfr pathway induces expression of Engrailed, a *scarface* repressor, in the lateral epidermis. (A, A’) Cuticle preparation. Dark (A) and bright field (A’) images of an embryo expressing *pnr>Yan*^*Act*^. Note the dorsal open hole. (B, C) Embryos expressing *pnr>Yan*^*Act*^, hybridized using a digoxigenin-labeled RNA probe for *dpp* (blue; B) or stained for pMad (red; C). (B’) Magnified view of the region marked by a rectangle in (B). Note that Yan^Act^ brings about a reduction in *dpp* expression and, as a consequence, a reduction in the pMad domain, similarly to other *Egfr* pathway mutants. (D) Embryo expressing *pnr>Yan*^*Act*^ hybridized using a digoxigenin-labeled RNA probe for *scaf* (blue). (D’) Magnified view of the region marked by a rectangle in (D). Note that *scaf* expression expands into the lateral epidermis. (E, F) Yan^ACT^ dominantly represses En. Control embryo expressing *pnr>GFP* (E) and embryo expressing *pnr>Yan*^*Act*^ (F) stained for En (green), as well as for LacZ (magenta; *puc-lacZ*) to mark the LE. Yan^ACT^ activity reduces En expression in the LacZ-positive LE cells, as well as in the adjacent lateral epidermis (F). (G) Model explaining how Egfr signaling prevents expression of *scaf* in the lateral epidermis.

We next addressed the mechanism by which Egfr signaling impacts on *scaf* expression. *scaf* could represent an exceptional example of a gene that is directly repressed by the Egfr pathway. Still, previous studies indicated that, at least in flies, RTK signaling pathways predominantly activate gene expression, often by downregulating negative transcriptional regulators such as the ETS transcription factor Yan (also known as Anterior open) [32]. Therefore, Egfr signaling could be affecting *scaf* expression indirectly by inducing an intermediary repressor of this gene. To begin testing this idea, we expressed a non-phosphorylatable derivative of Yan, Yan^Act^, that is insensitive to attenuation by Egfr-mediated signaling [33] and, hence, should dominantly block the induction of such a repressor. We find that *pnr>Yan*^*Act*^ embryos display dorsal open phenotypes (Fig 8A and 8A’; cf. Fig 2A), loss of *dpp* expression and reduced pMad staining (Fig 8B and 8C; cf. Fig 4A–4A”). Remarkably, *scaf* is derepressed in these embryos (Fig 8D; cf. Fig 6C), consistent with the idea that Yan normally represses a *scaf* repressor.

S. Noselli and colleagues recently demonstrated that *scaf* is under complex transcriptional control, involving several activators as well as at least two repressors: Engrailed (En) and Abdominal-A (Abd-A) [34]. They showed that in embryos mutant for *en*, *scaf* is derepressed only in LE cells, perhaps due to the combined repressor activity of Abd-A. Nevertheless, when they expressed a form of En that was converted into an activator, *scaf* was ectopically expressed in the lateral epidermis [34]. This raised the question whether the Egfr-Yan axis induces En, which in turn directly represses *scaf*. Indeed, we find that En is dominantly repressed in the lateral epidermis of embryos expressing *pnr>Yan*^*ACT*^, specifically in the *pnr* domain (Fig 8F; cf. 8E). Thus, a transcriptional regulatory cascade could explain how the Egfr pathway represses *scaf* (Fig 8G): by downregulating Yan, Egfr signaling induces expression of En (and perhaps other repressors) which, in turn, silences *scaf* expression. In embryos in which Egfr pathway activity is blocked, En is not induced and *scaf* is derepressed in the lateral epidermis, thereby hindering JNK signaling in LE cells.

## Discussion

Although extensively investigated, it is not fully understood how complex morphogenetic processes such as DC are controlled, and by which signaling pathways. In this manuscript we report that, in addition to the well-established JNK and Dpp pathways, signaling mediated by the Egfr is also instrumental to DC. We uncover a novel interplay between the Egfr and JNK pathways, specifically by demonstrating that Egfr signaling in the lateral epidermis suppresses the expression of the gene *scaf*, which encodes a proposed secreted JNK antagonist. Through this regulatory switch the Egfr pathway facilitates JNK signaling in LE cells (Fig 7G and 7H). Egfr signaling thus contributes cell non-autonomously to the expression of the JNK target *dpp*, to the phosphorylation of the downstream Dpp effector Mad and, consequently, to the synchronized morphogenetic movements orchestrated by Dpp signaling that are essential to successful DC.

It is currently unknown whether Scaf, the protein product of an established JNK pathway target gene [14,34–36], impinges on JNK pathway activity directly or indirectly. Scaf could be negatively regulating JNK signaling directly, by acting on an extracellular signal or on a putative receptor of the pathway [13]. However, it could also be playing a more general role, for example by degrading the extracellular matrix [35] or by establishing correct basement membrane protein localization [14], thus influencing JNK pathway outcomes indirectly. Although our study does not distinguish between these mechanisms, our results showing that Scaf impacts on the expression and activity of Dpp are consistent with the notion that Scaf is an antagonist of the JNK pathway. Rousset *et al*. reached similar conclusions, based on their findings that *scaf* loss-of-function mimics JNK over-activity as well as on other data [13]. Further research, however, will be required to conclusively elucidate the molecular mechanism(s) underlying Scaf function in the context of dorsal closure.

Our findings that the Egfr and JNK pathways are linked at the level of a JNK feedback inhibitor exemplify an important emerging theme in cell signaling: that Egfr signaling frequently impacts on the activity of other developmental pathways or master regulators via the induction of genes, whose protein products subsequently modulate the activity of these secondary pathways and/or factors. For instance, in the fly eye imaginal disc, Egfr signaling induces expression of the Delta ligand in photoreceptor cells, and thus positively stimulates Notch signaling in neighbouring cone cells [37,38]. In other cases, Egfr-regulated targets act as negative feedback regulators. For example, the Egfr pathway induces expression of the gene *wntD*, which encodes an antagonist of the Rel transcription factor, Dorsal. Through this negative feedback regulation, Egfr signaling opposes the nuclear localization of Dorsal, thereby affecting the expression of multiple Dorsal targets along the D/V axis of the embryo [39].

In our analyses, we have focused on the input to DC by Egfr signaling taking place in the lateral epidermis. It is conceivable that Egfr-mediated signal transduction also plays additional regulatory roles during DC. For example, this pathway has been previously implicated in the suppression of *zipper*, the gene encoding *Drosophila* non-muscle myosin II heavy chain, in the AS and in a cell non-autonomous manner also in LE cells [40]. Furthermore, our results do not preclude the involvement of additional RTK pathways in this developmental process. As a case in point, signaling by PVR in DC supports proper midline zippering in addition to AS internalization and removal, via the PI3K pathway and independently of JNK signaling [41]. Future studies will uncover the full impact of RTK-mediated signal transduction in DC.

In conclusion, our work illuminates a novel mechanism of signal integration between the Egfr and JNK pathways, linking Egfr signaling to the core regulatory network controlling DC. Our results thus reinforce the idea that different signaling pathways that regulate morphogenesis are interlinked, acting in a coordinated manner. A deeper understanding of the cross-regulation between these pathways, and the elucidation of further roles for Egfr signaling in DC, should facilitate our understanding of how diverse signal transduction pathways intersect to synchronize collective cell behavior, and how this circuitry ultimately leads to precise and coordinated morphogenetic processes.

## Materials and methods

### Fly culture and stocks

Flies were cultured and crossed on standard yeast-cornmeal-molasses-malt extract-agar medium at 25°C. The following mutant stocks and Gal4 drivers were used: *Egfr*^*EA*^, *Egfr*^*EB*^ [31], *scaf*^*Δ1*.*5*^, *UAS-Scaf* [14], *rhomboid*^*7M*^, *spi*^2A14^, *UAS-Egfr*^*DN*^, *UAS-Ras*^*DN*^, *UAS-Ras*^*V12*^, *bsk*^*1*^, *UAS-Bsk*^*DN*^, *UAS-Hep*^*Act*^, *UAS-Yan*^*Act*^ and *pnr-Gal4*, *puc-lacZ* (*puc*^*E69*^). In general, mutant chromosomes were maintained over *wg-LacZ*- or *dfd-YFP*-marked balancer chromosomes, allowing the unambiguous identification of embryos of the correct genotype. *yellow white* flies served as wild-type controls.

To quantify the proportion of embryos compromised in Egfr signaling that fail to complete closure at st16, the following genotypes were used: 1) *white; pnr*-*Gal4*, *puc-lacZ/UAS-GFP* (control); 2) *white; UAS-Egfr*^*DN*^*; pnr*-*Gal4*, *puc-lacZ*; 3) and *UAS-Ras*^*DN*^*; pnr*-*Gal4*, *puc-lacZ*.

### Cuticle preparation

Unhatched larvae (>24 hours old) were dechorionated in bleach, devitellinised in 1:1 methanol/heptane, rehydrated in PBS/methanol and mounted in 1:1 Hoyer’s medium/double-distilled water and cleared overnight at 70°C.

### *In situ* hybridization and antibody staining

Embryos were dechorionated in bleach and fixed in 8% formaldehyde/PBS/heptane for 20 minutes. Expression patterns of *dpp* and *scaf* were visualized by whole-mount *in situ* hybridization using digoxigenin-UTP labeled antisense RNA probes and anti-digoxigenin antibodies conjugated to alkaline phosphatase (Roche).

Fluorescent immunodetection of dpErk, in freshly fixed embryos (10% formaldehyde/PBS/Heptane buffer), was attained using rabbit αdpErk (1:100; Cell Signaling) [20]. Other antibodies used were: mouse αLacZ (1:1000; Promega), rabbit αLacZ (1:2000; Cappel), rabbit αpMad (1:100; Epitomics), mouse αEn (1:20; Developmental Studies Hybridoma Bank) and rat αDE-Cadherin (1:50; Developmental Studies Hybridoma Bank). Secondary antibodies were Alexa Fluor 488-, 546-, and 633-conjugated (1:400; Jackson Laboratories). Embryos were mounted using Vectashield medium (Vector Laboratories).

### Microscopy

Light microscope images were acquired using a Zeiss Axioplan2 microscope and confocal images were taken using a Zeiss LSM710 confocal microscope. Images were processed using Adobe Photoshop software, and the ZEN 2012 blue edition was used to measure LE cell length in embryos compromised in Egfr signaling.

## Supporting information

S1 Fig *pannier*-Gal4 drives ectodermal GFP expression throughout the lateral epidermis and LE cells. (A-C) A *pnr*-Gal4>GFP, *puc*-*lacZ* enhancer-trap embryo, stained for LacZ (red; A) and for GFP (green; B). (C) Merge. Note that ectodermal expression of GFP, driven by *pnr*-Gal4, is restricted to the lateral epidermis and LE cells.
(TIF)

S2 Fig Quantifying incomplete dorsal closure in embryos defective in Egfr signaling. (A-B) Confocal images of st16 *puc*-*lacZ* enhancer-trap line embryos, in which *pnr*-Gal4 drives the expression either of *GFP* (control), *Ras*^*DN*^ or *Egfr*^*DN*^, stained for LacZ to demarcate LE cells. The numbers of st16 embryos, displaying a dorsal-open hole and therefore incomplete closure (A; red), or those that have completed closure (B; blue), were scored. (C) Percentage of st16 embryos, expressing *GFP*, *Ras*^*DN*^ or *Egfr*^*DN*^ via *pnr*-Gal4, that have completed closure (blue) or not (red). n = number of embryos from each definitive genotype that were scored.
(TIF)

S3 Fig Egfr pathway activity is required for proper LE cell elongation. Quantification of LE cell length in wild-type or in *bsk*, *spi* and *rhomboid* mutant embryos, as well as in embryos expressing *pnr>Egfr*^*DN*^ or *pnr>Ras*^*DN*^. The data represent the mean ± SD derived from 8–10 different embryos. *** P<0.0001 compared to wild-type embryos (Mann-Whitney U-test). n = number of LE cells from each definitive genotype that were scored.
(TIF)

S4 Fig Egfr signaling is required for the full expression of *dpp*. (A-D) High magnification (x40) lateral views of embryos hybridized using a digoxigenin-labeled RNA probe for *dpp* (blue). Small insets show the full embryos. (A) Wild-type embryo showing the normal *dpp* pattern. Levels of *dpp* are reduced in a *rhomboid* mutant (B) as well as in embryo expressing *pnr*>*Egfr*^*DN*^ (C). Conversely, the *dpp* domain expands ventrally in embryo expressing *pnr*>*Ras*^*V12*^ (D).
(TIF)

S5 Fig Expression of *Egfr*^*DN*^ in stripes leads to defective dorsal closure. (A) Cuticle preparation of embryo expressing *prd>Egfr*^*DN*^ showing an open dorsal phenotype (white arrowhead). (B) St13 embryo expressing *prd>Egfr*^*DN*^ hybridized using a digoxigenin-labeled RNA probe for *dpp* (blue). Loss of *dpp* expression (black arrowheads) in both stripe and inter-stripe regions of *prd>Egfr*^*DN*^ embryos indicates that the resulting ectopic Scaf acts on LE cells non- autonomously (see below). White asterisks and arrowheads mark JNK-independent *dpp* expression in the visceral mesoderm and lateral ectoderm, respectively.
(TIF)

S6 Fig Egfr pathway perturbations primarily affect the lateral epidermis. Control *puc-lacZ*, *pnr*-Gal4 embryo expressing GFP (A), or *puc-lacZ*, *pnr*-Gal4 embryos expressing Egfr^DN^ (B) and Ras^DN^ (C), stained for pMad (red) and LacZ (blue). Note that LE cells, distinguishable by LacZ staining, co-stain for pMad, whereas pMad staining is markedly reduced in the lateral epidermis.
(TIF)

S7 Fig *Egfr loss-of-function* from stage 12 onwards leads to dorsal closure defects. (A, B) Cuticle preparations of embryos carrying the temperature sensitive *Egfr*^*SH2*^ allele, maintained at permissive (18°C) (A) or restrictive (29°C) (B) temperatures. The embryo in (B) was shifted from the permissive to the restrictive temperature at the onset of dorsal closure (st12). Note the dorsal open phenotype (arrowhead). Wild-type embryos subjected to the same regime hatched normally. The embryo in (A) has mild segmental defects. (C, D) The domain of pMad staining (red) decreases in *Egfr*^*SH2*^ embryo shifted to 29°C at st12 (D) but not in embryo of the same genotype raised at 18°C (C). (E, F) Embryos stained for DE-cadherin (green) to outline cell membranes. Corresponding primed panels (E’ and F’) show magnified views of the regions marked with arrowheads. Note the occurrence of cell elongation defects in F’.
(TIF)

S8 Fig The loss of pMad staining in *pnr>Bsk*^*DN*^*;Ras*^*V12*^ embryos is not due to Gal4 dilution. (A-F) Lateral views of st13 embryos stained for GFP (green) and pMad (red). (A-C) Control embryo, expressing *pnr>GFP*, stained for (A) GFP and (B) pMad. (C) Merge. (D-F) Embryo co-expressing *pnr>Ras*^*V12*^*;GFP*, stained for (D) GFP and (E) pMad. (F) Merge. Note the strong pMad staining in *pnr>Ras*^*V12*^*;GFP* embryo.
(TIF)

S9 Fig The dpErk pattern is unaltered in embryos with loss- or gain-of-function JNK signaling. (A-C) Embryos stained for dpErk (red). No significant change in the dpErk pattern is observed in *bsk* mutant embryo (B) or upon *pnr>Hep*^*Act*^ expression (C), compared to control (A). The signal in the AS is an artifact caused by auto-florescence.
(TIF)
