# Supplementary figures and images for: Novel interplay between JNK and Egfr signaling in Drosophila dorsal closure
Source: PLoS Genet. 2017 Jun 19;13(6):e1006860. doi: 10.1371/journal.pgen.1006860 (PMC5495517; doi:10.1371/journal.pgen.1006860)

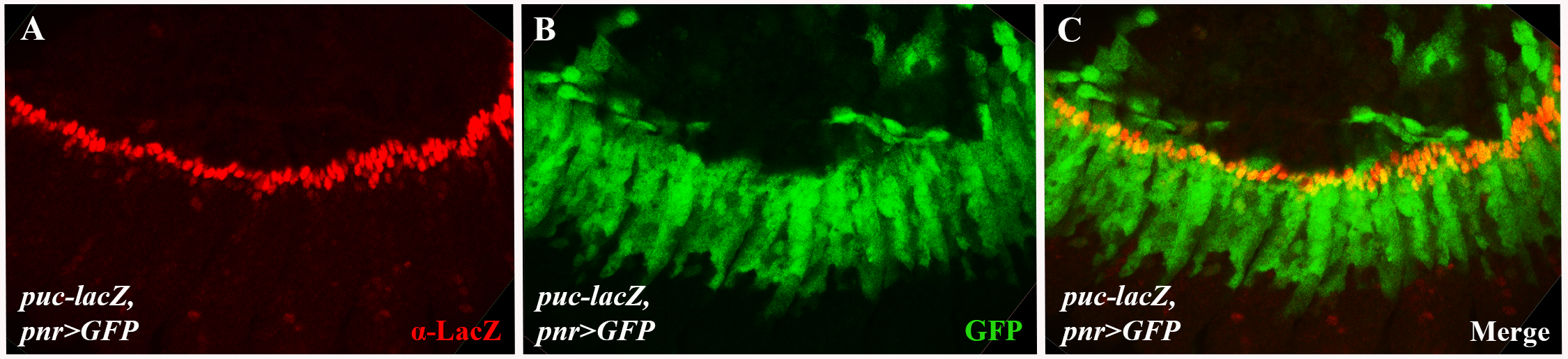

Supplement: S1 Fig — (A-C) A pnr-Gal4>GFP, puc-lacZ enhancer-trap embryo, stained for LacZ (red; A) and for GFP (green; B). (C) Merge. Note that ectodermal expression of GFP, driven by pnr-Gal4, is restricted to the lateral epidermis and LE cells. (TIF) [file pgen.1006860.s001.tif]

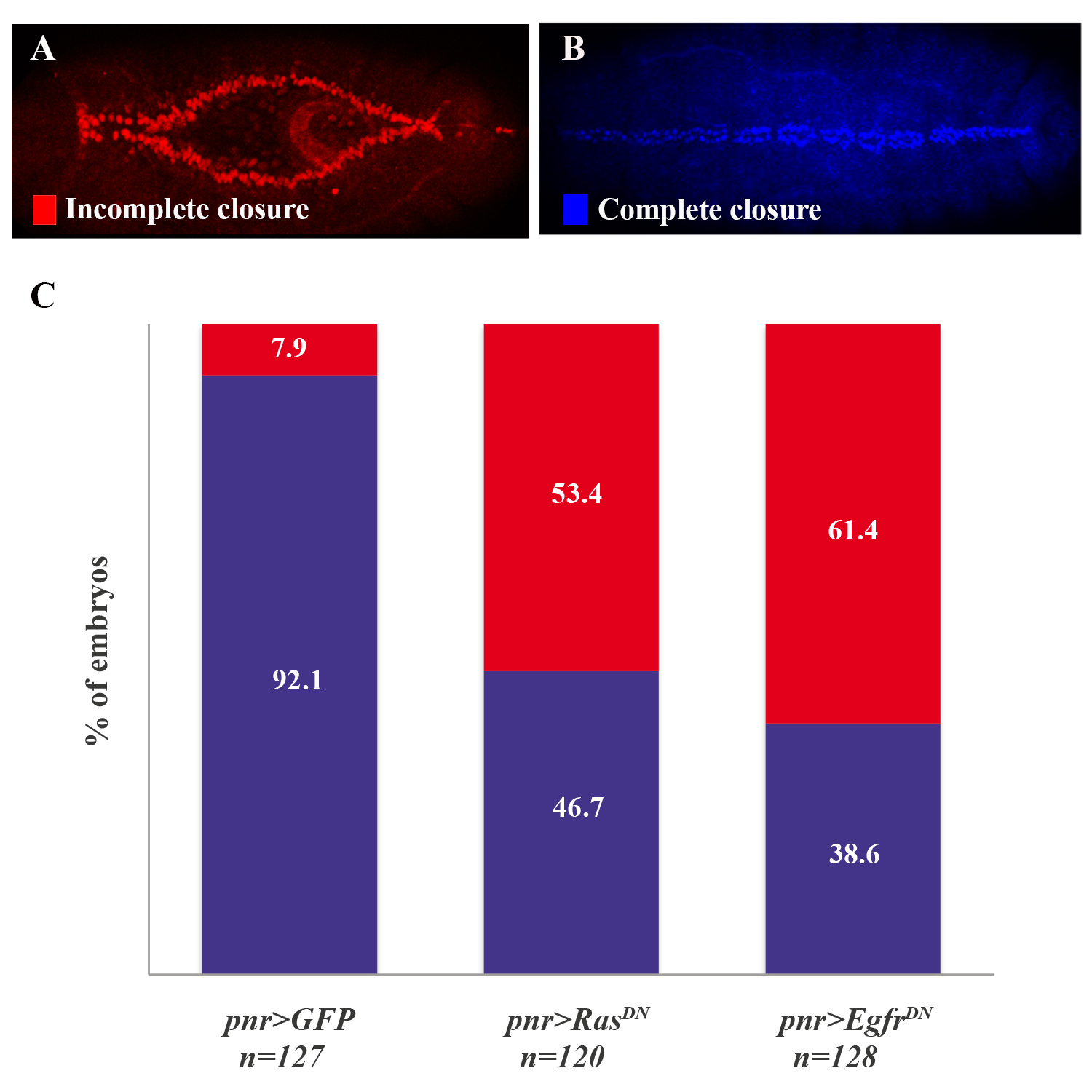

Supplement: S2 Fig — (A-B) Confocal images of st16 puc-lacZ enhancer-trap line embryos, in which pnr-Gal4 drives the expression either of GFP (control), RasDN or EgfrDN, stained for LacZ to demarcate LE cells. The numbers of st16 embryos, displaying a dorsal-open hole and therefore incomplete closure (A; red), or those that have completed closure (B; blue), were scored. (C) Percentage of st16 embryos, expressing GFP, RasDN or EgfrDN via pnr-Gal4, that have completed closure (blue) or not (red). n = number of embryos from each definitive genotype that were scored. (TIF) [file pgen.1006860.s002.tif]

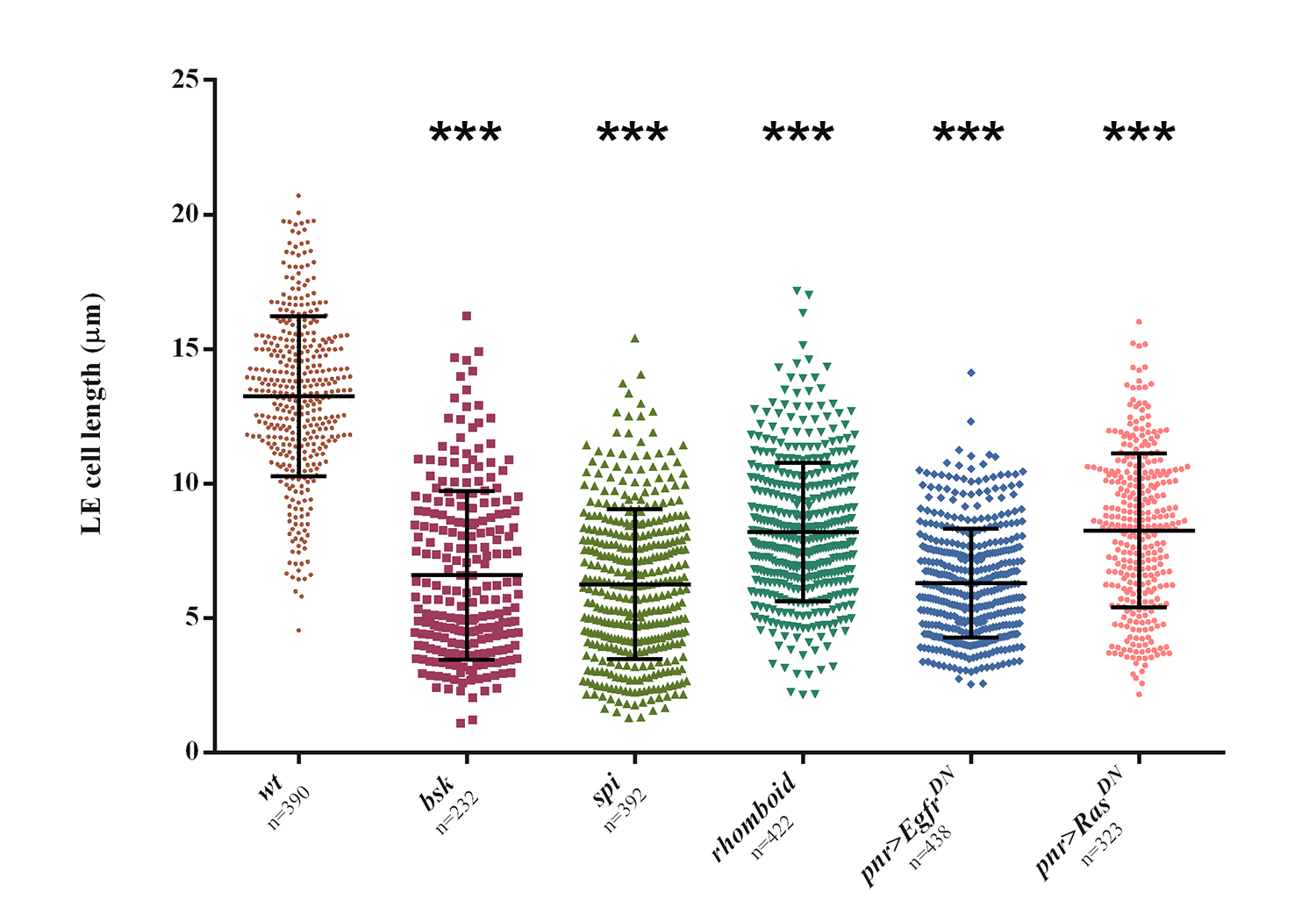

Supplement: S3 Fig — Quantification of LE cell length in wild-type or in bsk, spi and rhomboid mutant embryos, as well as in embryos expressing pnr>EgfrDN or pnr>RasDN. The data represent the mean ± SD derived from 8–10 different embryos. *** P<0.0001 compared to wild-type embryos (Mann-Whitney U-test). n = number of LE cells from each definitive genotype that were scored. (TIF) [file pgen.1006860.s003.tif]

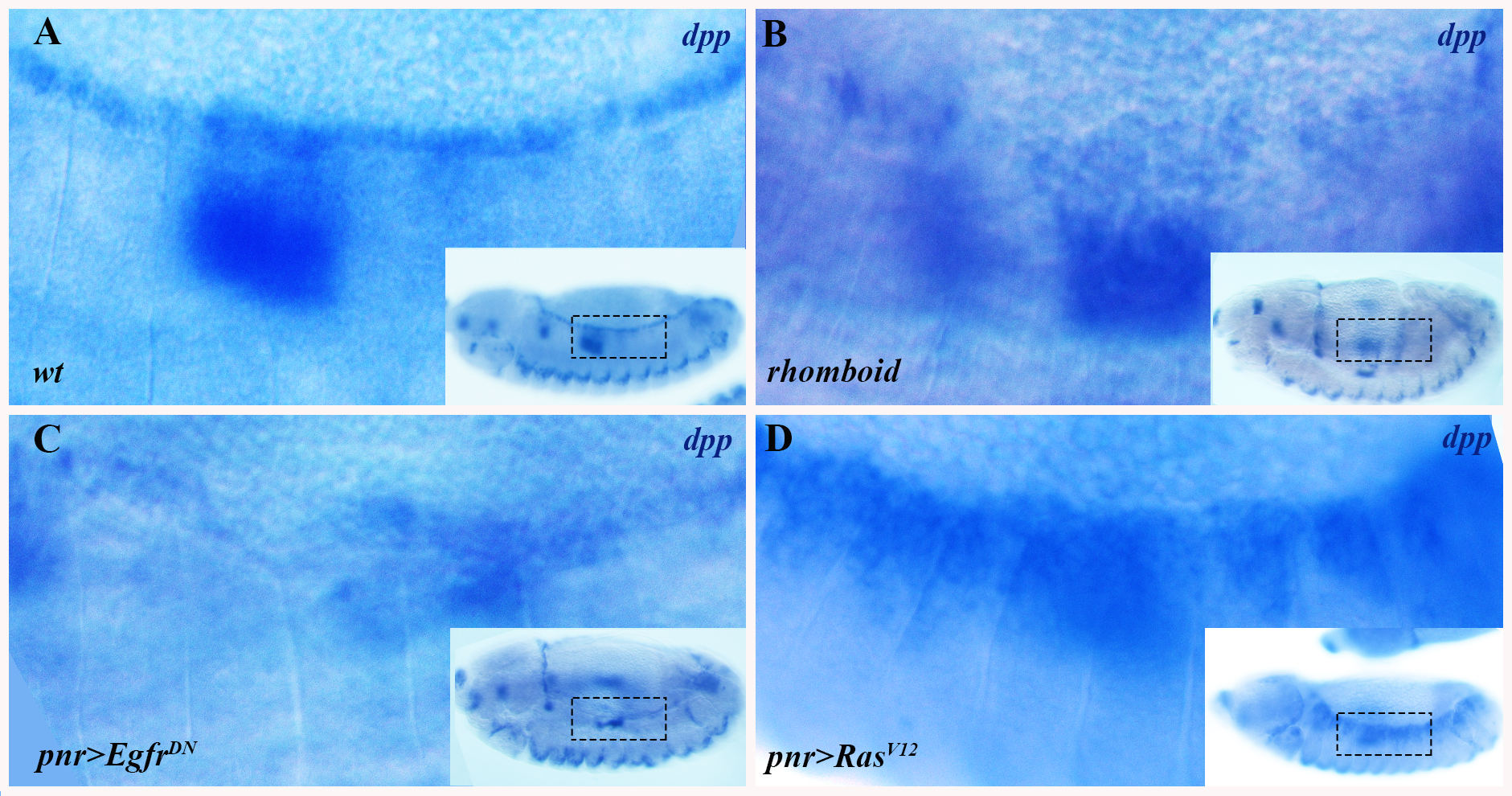

Supplement: S4 Fig — (A-D) High magnification (x40) lateral views of embryos hybridized using a digoxigenin-labeled RNA probe for dpp (blue). Small insets show the full embryos. (A) Wild-type embryo showing the normal dpp pattern. Levels of dpp are reduced in a rhomboid mutant (B) as well as in embryo expressing pnr>EgfrDN (C). Conversely, the dpp domain expands ventrally in embryo expressing pnr>RasV12 (D). (TIF) [file pgen.1006860.s004.tif]

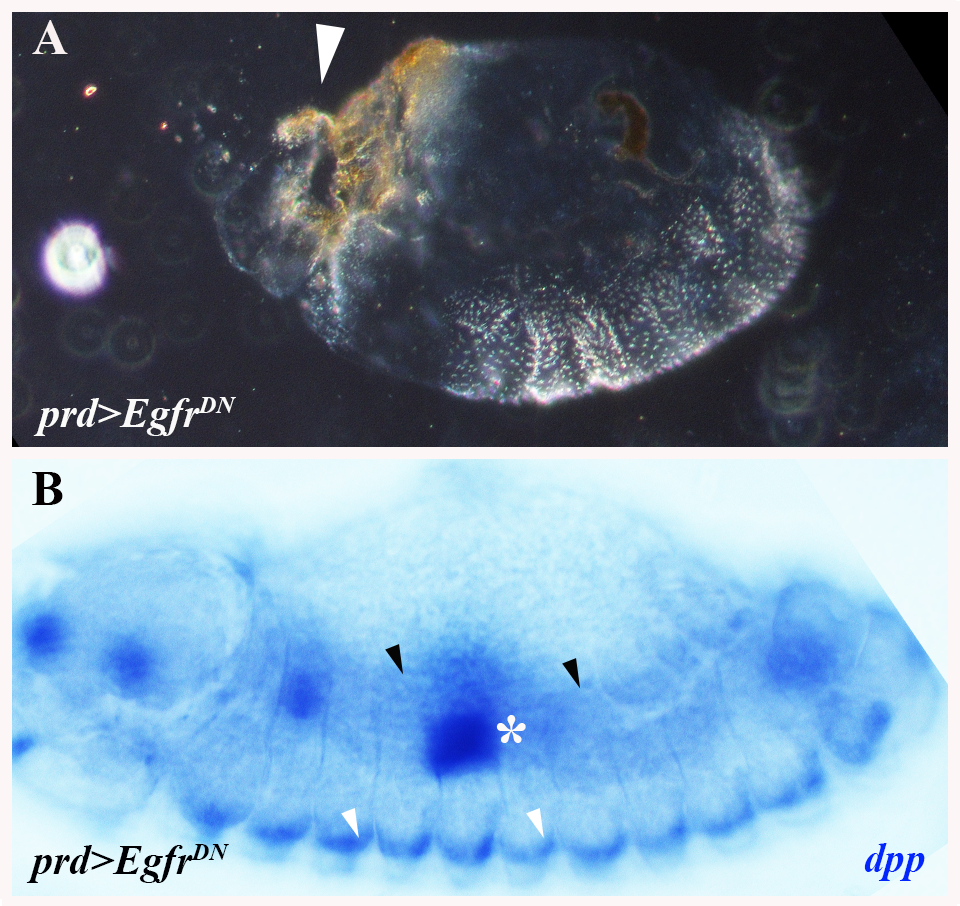

Supplement: S5 Fig — (A) Cuticle preparation of embryo expressing prd>EgfrDN showing an open dorsal phenotype (white arrowhead). (B) St13 embryo expressing prd>EgfrDN hybridized using a digoxigenin-labeled RNA probe for dpp (blue). Loss of dpp expression (black arrowheads) in both stripe and inter-stripe regions of prd>EgfrDN embryos indicates that the resulting ectopic Scaf acts on LE cells non- autonomously (see below). White asterisks and arrowheads mark JNK-independent dpp expression in the visceral mesoderm and lateral ectoderm, respectively. (TIF) [file pgen.1006860.s005.tif]

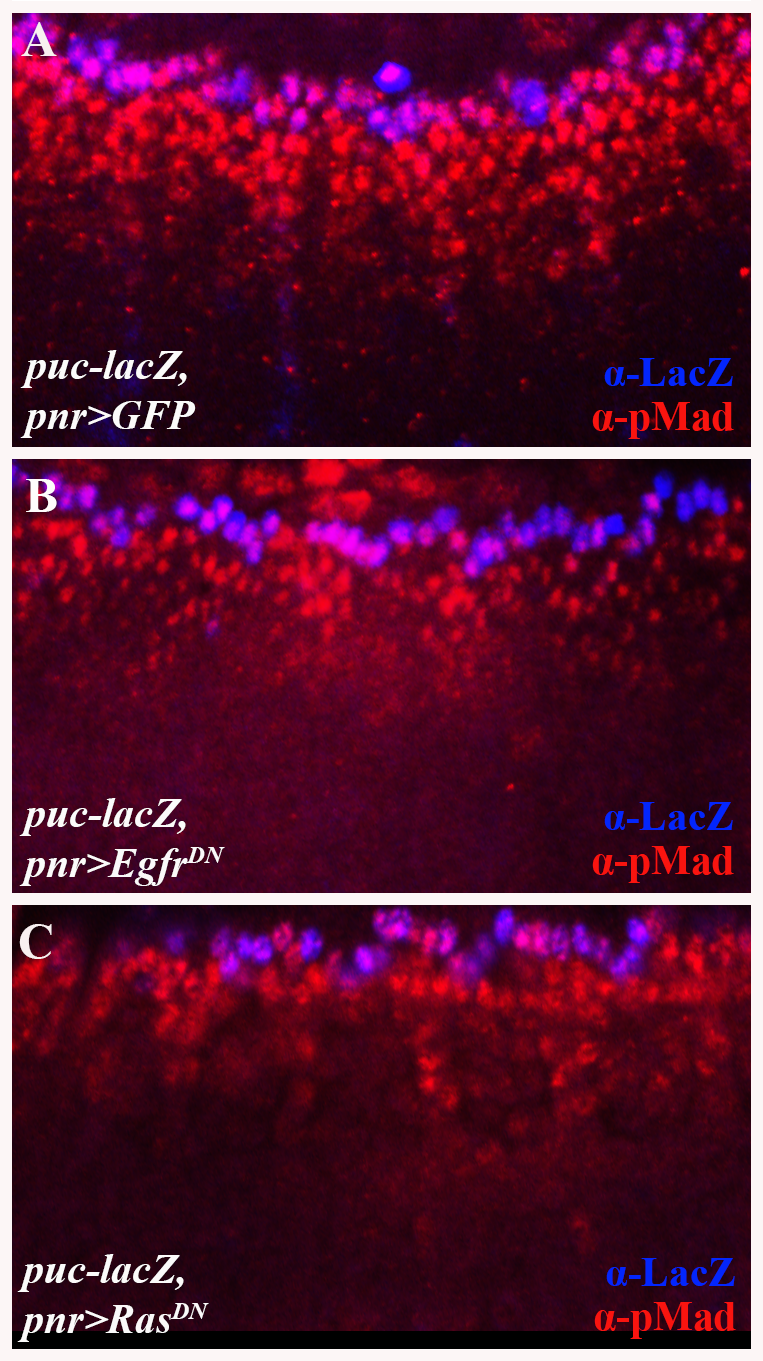

Supplement: S6 Fig — Control puc-lacZ, pnr-Gal4 embryo expressing GFP (A), or puc-lacZ, pnr-Gal4 embryos expressing EgfrDN (B) and RasDN (C), stained for pMad (red) and LacZ (blue). Note that LE cells, distinguishable by LacZ staining, co-stain for pMad, whereas pMad staining is markedly reduced in the lateral epidermis. (TIF) [file pgen.1006860.s006.tif]

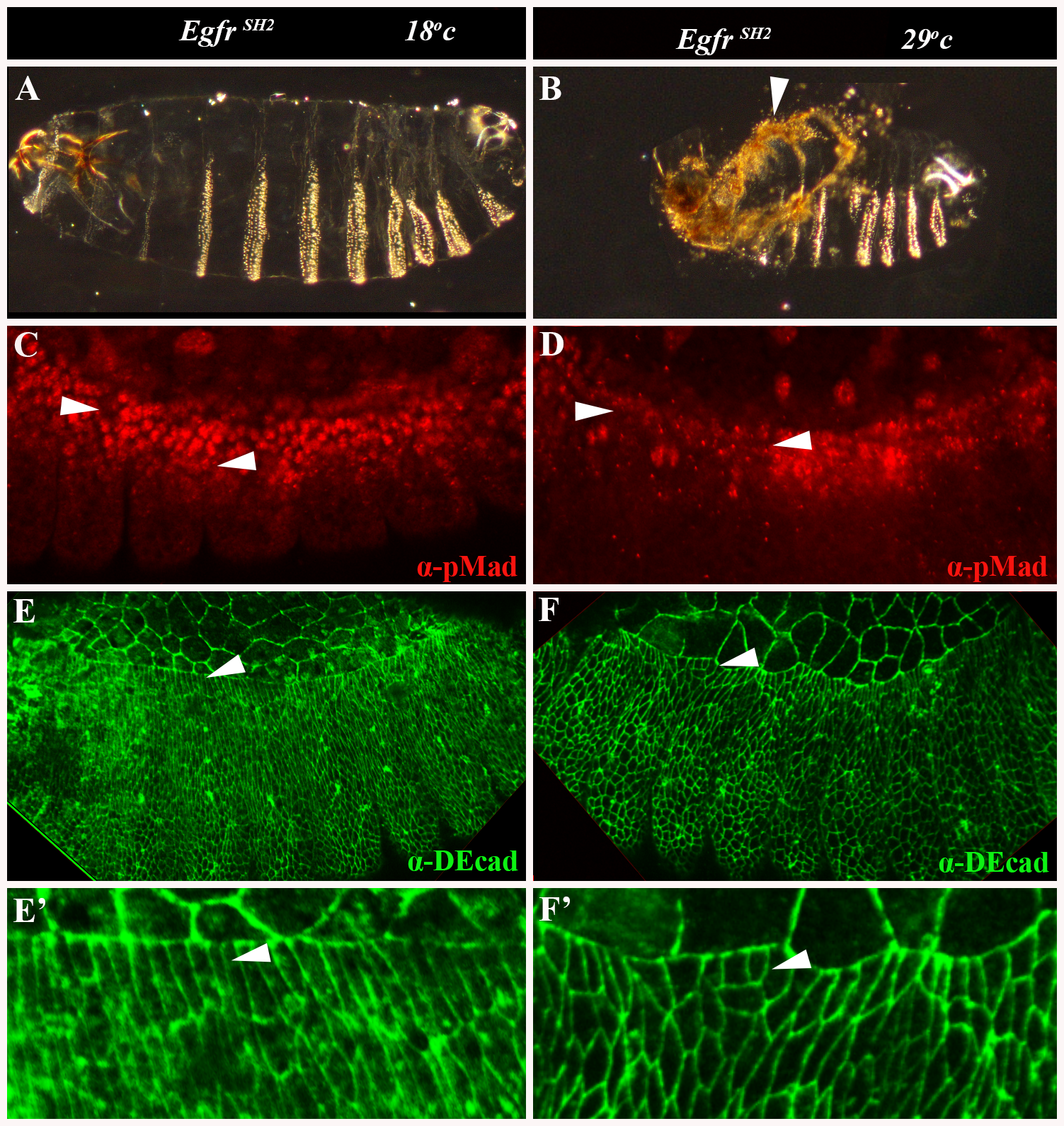

Supplement: S7 Fig — (A, B) Cuticle preparations of embryos carrying the temperature sensitive EgfrSH2 allele, maintained at permissive (18°C) (A) or restrictive (29°C) (B) temperatures. The embryo in (B) was shifted from the permissive to the restrictive temperature at the onset of dorsal closure (st12). Note the dorsal open phenotype (arrowhead). Wild-type embryos subjected to the same regime hatched normally. The embryo in (A) has mild segmental defects. (C, D) The domain of pMad staining (red) decreases in EgfrSH2 embryo shifted to 29°C at st12 (D) but not in embryo of the same genotype raised at 18°C (C). (E, F) Embryos stained for DE-cadherin (green) to outline cell membranes. Corresponding primed panels (E’ and F’) show magnified views of the regions marked with arrowheads. Note the occurrence of cell elongation defects in F’. (TIF) [file pgen.1006860.s007.tif]

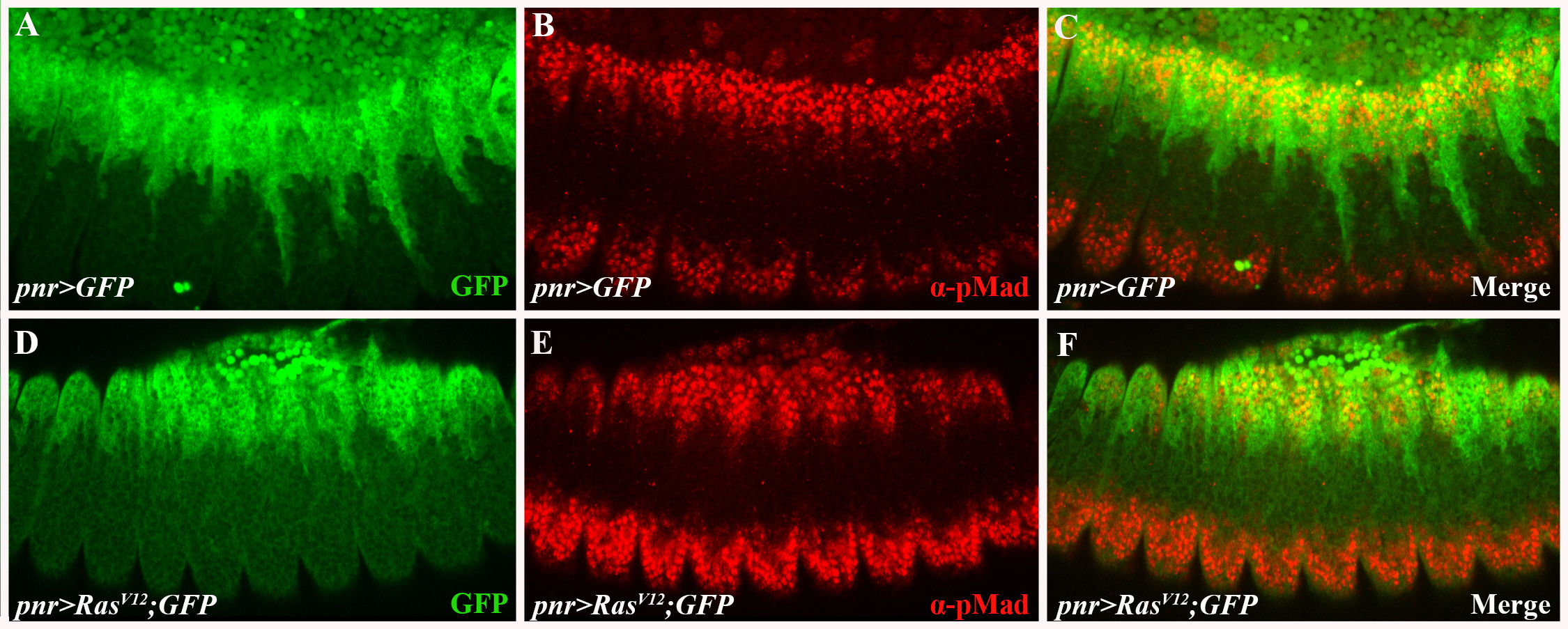

Supplement: S8 Fig — (A-F) Lateral views of st13 embryos stained for GFP (green) and pMad (red). (A-C) Control embryo, expressing pnr>GFP, stained for (A) GFP and (B) pMad. (C) Merge. (D-F) Embryo co-expressing pnr>RasV12;GFP, stained for (D) GFP and (E) pMad. (F) Merge. Note the strong pMad staining in pnr>RasV12;GFP embryo. (TIF) [file pgen.1006860.s008.tif]

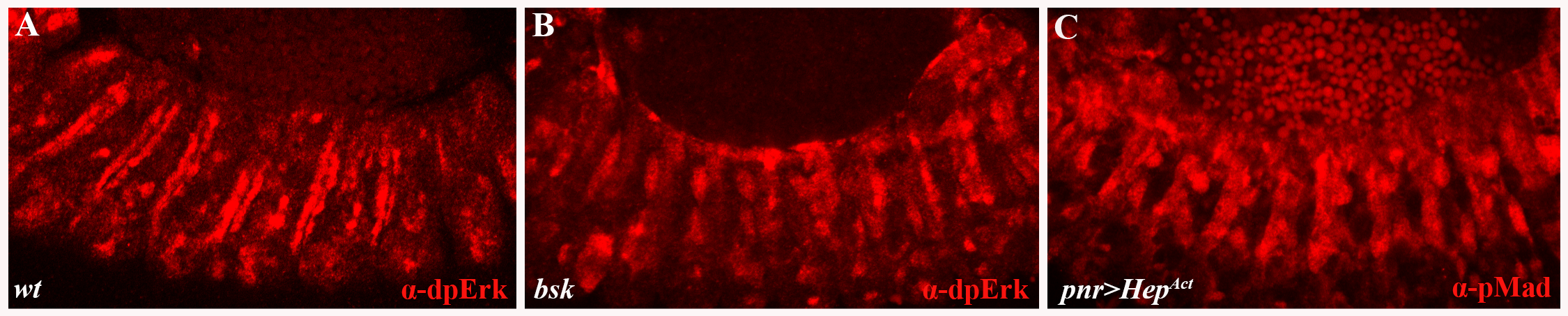

Supplement: S9 Fig — (A-C) Embryos stained for dpErk (red). No significant change in the dpErk pattern is observed in bsk mutant embryo (B) or upon pnr>HepAct expression (C), compared to control (A). The signal in the AS is an artifact caused by auto-florescence. (TIF) [file pgen.1006860.s009.tif]
